# Changes in Spinal Alignment following eXtreme Lateral Interbody Fusion Alone in Patients with Adult Spinal Deformity using Computed Tomography

**DOI:** 10.1038/s41598-019-48539-w

**Published:** 2019-08-19

**Authors:** Akihiko Hiyama, Hiroyuki Katoh, Daisuke Sakai, Masato Sato, Masahiro Tanaka, Tadashi Nukaga, Masahiko Watanabe

**Affiliations:** Department of Orthopaedic Surgery, Tokai University School of Medicine, 143 Shimokasuya, Isehara, Kanagawa 259-1193 Japan

**Keywords:** Neurological disorders, Neurodegenerative diseases

## Abstract

This study examined the ability of the extreme lateral interbody fusion (XLIF) procedure to restore coronal and sagittal alignments for patients with adult spinal deformity (ASD) using computed tomography multiplanar reconstruction (CT-MPR). Thirty-eight patients with ASD undergoing correction and fixation with XLIF at 114 levels were studied. The coronal segmental Cobb angle, coronal regional Cobb angle (L1-5), sagittal segmental Cobb angle, sagittal regional Cobb angle (L1-5), intervertebral disc height and, vertebral body rotation (VBR) were measured before and after of XLIF surgery using CT-MPR. The mean sagittal segmental Cobb angle, the coronal segmental Cobb angle and VBR were corrected from 5.0° to 9.0°, from 6.3° to 4.3° and from 12.2° to 10.8°, respectively. The mean of the intervertebral disc heights increased significantly from 6.0 mm to 10.4 mm postoperatively. Although increases in coronal segmental Cobb, sagittal segmental Cobb, and intervertebral disc height at each level were significant, there were no significant differences in each parameter acquired by spine levels. The results also showed that it was difficult for L4/5 level to obtain the most postoperative coronal Cobb, sagittal Cobb and intervertebral disc height. This study evaluated the alignment improvement effect of stand-alone XLIF in ASD patients using CT-MPR. For the lower lumbar spine, it is difficult to obtain a lordosis more than 10 degrees with stand-alone XLIF for correcting ASD. Therefore, it is thought that correction such as osteotomy or compression technique to the posterior fusion may be necessary during the 2^nd^ stage surgery.

## Introduction

Adult spinal deformity (ASD) is defined as an angular deformity presenting after skeletal maturity, and can be divided into coronal (scoliosis), sagittal (kyphosis), and mixed^1^. With the sagittal alignment of the spine being one of the most important factors influencing disorders of lower back, the surgical correction of ASD is meant to restore spinal balance and improve the patient’s health-related quality of life (QOL)^2–4^. Traditional approaches to surgically correct ASD include anterior-posterior approaches or, more commonly, posterior-only approaches^5^. Moreover, more rigid and severe deformities may need a combined anterior-posterior approach or a posterior three-column reconstruction technique to obtain correction of spinal alignment. However, ASD surgery remains extremely challenging, and these procedures are invasive for the elderly^6^.

The extreme lateral interbody fusion (XLIF) technique, which uses a transpsoas retroperitoneal approach, has been popularized as a minimally invasive alternative surgical option for anterior column reconstruction and arthrodesis^7^. One of the advantages of XLIF is that a wide interbody cage can be inserted into the narrowed intervertebral disc space, correcting the scoliosis and achieving stable fixation. Therefore, XLIF has been reported to achieve good clinical and radiological results when employed in the correction surgery for ASD patients^8,9^. XLIF has also been shown to be safe in elderly patients and is said to be superior to open transforaminal lumbar interbody fusion (TLIF) historical controls in terms of complication rate, blood loss, and transfusion rate^10^. To date, numerous radiographic studies have shown significant improvement in foraminal height, intervertebral disc height, and the cross-sectional area with indirect decompression through the XLIF procedure. Multicenter studies on the XLIF procedure have reported improvements in coronal segmental angles, segmental lordosis, and disc height^11–13^. According to formulas for ASD correction, the restoration of lumbar lordosis is considered to be most important. Since L4- S1 lordosis accounts for 2/3 of the total lumbar lordosis, emphasis is placed on restoring lordosis in the lower lumbar levels^14^.

Recently, it has been reported that significant improvements in sagittal and coronal alignments can be obtained in correction surgery for ASD patients by using a two-stage combined approach^15^. However, most past studies have evaluated alignment after posterior spinal surgery and not lateral lumbar interbody fusion (LLIF) alone^16,17^. Therefore, an analysis on the corrective capability of LLIF alone is important to consider if osteotomies are necessary in addition to segmental LLIF fixation to achieve the planned correction. The purpose of this study is to evaluate the spinal alignments of ASD patients treated by XLIF alone using computed tomography multiplanar reconstruction (CT-MPR) immediately after the first stage of surgery.

## Results

The spine-pelvic parameters in the preoperative standing full-length x-rays of the 38 patients are shown in Table 1.

**Table 1:** Detailed coronal and sagittal plane parameters of the subjects. CR Cobb: Coronal Cobb; TK: Thoracic kyphosis angle (T5–12); LL: Lumbar lordosis angle (L1–S1); SS: Sacral slope; PI: Pelvic incidence; PT: Pelvic tilt; SVA: Sagittal vertical axis. All values are in mean ± standard deviation.

**ASD (n** = **38)**	
CR Cobb (°)	38.6 ± 18.1
TK (°)	28.7 ± 21.3
LL (°)	10.1 ± 24.0
SS (°)	19.6 ± 10.6
PI (°)	51.7 ± 9.2
PT (°)	32.9 ± 8.0
SVA (mm)	170.9 ± 72.9

As seen in Table 2, the mean sagittal segmental Cobb angle measured 5.0° preoperatively, and 9.0° postoperatively (p < 0.001). The mean preoperative regional lumbar lordosis angle increased from 27.0° (range, kyphosis 7.1° to lordosis 78.1°) to 36.0° (range, lordosis 9.9° to 78.5°). The mean coronal segmental Cobb angle was 6.3° preoperatively and 4.3° postoperatively (p < 0.01). The mean pre- and postoperative regional lumbar coronal Cobb angles were 16.0° and 12.6°, respectively (p = 0.05). The mean disc height was 6.0 mm preoperatively, and 10.4 mm postoperatively (p < 0.001). The ⊿ sagittal segmental Cobb angle, ⊿ coronal segmental Cobb angle, and ⊿ disc height were 4.0 ± 5.2°, 2.0 ± 4.1°, 4.4 ± 2.5 mm, respectively.

**Table 2:** Radiological outcomes. All values are in mean ± standard deviation.

	Pre XLIF	Post XLIF	p value
Coronal regional Cobb (L1-5) (°)	16.0 ± 13.5	12.6 ± 11.5	<0.05
Regional lumbar lordosis (L1-5) (°)	27.0 ± 19.4	36.0 ± 16.5	<0.001
Coronal segmental Cobb (°)	6.3 ± 4.4	4.3 ± 3.4	<0.01
Sagittal segmental Cobb (°)	5.0 ± 3.9	9.0 ± 4.6	<0.001
Interveretebral disc height (mm)	6.0 ± 2.2	10.4 ± 2.0	<0.001

XLIF was performed for a total of 114 levels in the 38 patients as follows: 1-level procedure (n = 1), 2-level (n = 10), 3-level (n = 15), and 4-level (n = 12). The position of the XLIF cage was 40.6 ± 14.0%, and from the front of less than 60% to the center were 105 levels (92.1%). Regarding the position of the XLIF cages, there was a tendency for the cages to be placed more posteriorly as the levels descended through L1/2 to L4/5, but there was no statistically significant difference between spinal levels (data not shown).

We compared the results between the 10 degrees cages (10°, n = 88 levels) and 15 degrees cages (15°, n = 26 levels), but cage obliquity was not significantly associated with scoliosis, lordosis, or disc height change (Table 3).

**Table 3:** Influence of XLIF on cage obliquity. All values are in mean ± standard deviation.

114 levels	Cage obliquity		
	10°(n = 88)	15°(n = 26)	p value
⊿ Coronal segmental Cobb (°)	−2.2 ± 4.3	−1.3 ± 3.2	0.632
⊿ Sagittal segmental Cobb (°)	3.9 ± 5.2	4.4 ± 4.8	0.690
⊿ Interveretebral disc height (mm)	4.5 ± 2.6	4.0 ± 2.0	0.277

**Figure 1:**
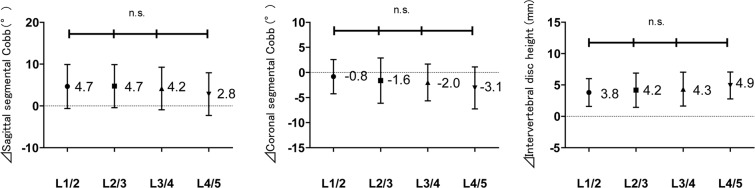
Changes in radiographical indices according to spinal level: ⊿ sagittal segmental Cobb angle, ⊿ coronal segmental Cobb angle, and ⊿ intervertebral disc height n.s; no significant difference between levels (P > 0.05). The numbers represent the average.

The segmental alignment after the XLIF surgery showed that it was most smallest for the L4/5 level to gain postoperative coronal Cobb, sagittal Cobb, and intervertebral disc height. Although increases in coronal segmental Cobb, sagittal segmental Cobb, and intervertebral disc height at each level were significant (Table 4), ⊿ sagittal segmental Cobb angle, ⊿ coronal segmental Cobb angle, and ⊿ disc height were not statistically significant by spinal levels (Fig. 1).

**Table 4:** Change in segmental Cobb and disc height by spinal level. All values are in mean ± standard deviation.

	Spine levels	Number	Pre XLIF	Post XLIF	p value
Coronal segmental Cobb (°)	L1/2	15	5.0 ± 3.7	4.2 ± 3.2	0.359
	L2/3	34	6.8 ± 4.5	5.2 ± 3.9	<0.05
	L3/4	35	6.4 ± 4.2	4.4 ± 3.3	<0.01
	L4/5	30	6.4 ± 5.1	3.3 ± 2.8	<0.001
Sagittal segmental Cobb (°)	L1/2	15	5.9 ± 4.7	10.5 ± 4.5	<0.01
	L2/3	34	4.9 ± 3.6	9.6 ± 4.2	<0.001
	L3/4	35	4.3 ± 4.0	8.5 ± 4.4	<0.001
	L4/5	30	5.4 ± 3.7	8.2 ± 5.3	<0.01
Interveretebral disc height (mm)	L1/2	15	6.9 ± 2.2	10.7 ± 2.2	<0.001
	L2/3	34	7.1 ± 2.4	11.2 ± 2.0	<0.001
	L3/4	35	5.7 ± 2.1	10.1 ± 1.8	<0.001
	L4/5	30	4.8 ± 1.6	9.7 ± 1.8	<0.001

In addition, the mean vertebral body rotation (VBR) was 12.2 ± 9.4° preoperatively and 10.8° ± 8.9° postoperatively (p < 0.001). When compared to the spine levels, we found that there was no statistically significant difference between spine levels (Table 5).

**Table 5:** Change in vertebral body rotation by spine levels. All values are in mean ± standard deviation.

	Spine levels	Pre XLIF	Post XLIF	p value
Vertebral body rotation (VBR)	L1	13.8 ± 10.0	12.7 ± 9.4	0.193
	L2	16.1 ± 10.4	14.6 ± 10.1	0.099
	L3	14.4 ± 10.3	13.1 ± 9.2	0.177
	L4	9.9 ± 7.0	8.5 ± 7.2	0.059
	L5	6.7 ± 5.1	5.1 ± 4.0	0.114

VBR: Vertebral body rotation.

Analysis of the correlation between ⊿ sagittal segmental Cobb and cage position of the 114 levels showed a negative weak correlation (r = −0.269, *P* < 0.001). The correlation between ⊿ sagittal segmental Cobb and pre-sagittal segmental Cobb (r = −0.486, *P* < 0.001) and pre-intervertebral disc height (r = −0.251, *P* < 0.01) were found (Table 6). That is, the smaller the sagittal segmental Cobb and intervertebral disc height were before XLIF surgery, the greater the increase in sagittal segmental Cobb obtained through surgery.

**Table 6:** Spearman correlations mean (Spearman’s *r*) between ⊿SSC and radiological parameters SSC; Sagittal segmental Cobb, CSC; Coronal segmental Cobb. IDH; Intervertebral disc height.

	⊿ SSC	⊿ CSC	⊿ IDH	Pre SSC	Post SSC	Pre CSC	Post CSC	Pre IDH	Post IDH
⊿SSC	1.000	−0.105	0.234*	−0.486***	0.690	0.097	0.030	−0.251**	0.089
⊿CSC	−0.105	1.000	−0.162	0.062	−0.064	−0.660***	0.253**	0.213*	0.043
⊿IDH	0.234*	−0.162	1.000	−0.320**	0.014	0.217*	0.059	−0.637***	0.511***
Pre SSC	−0.486***	0.062	−0.320**	1.000	0.249**	−0.171	−0.122	0.529***	0.140
Post SSC	0.690	−0.064	0.014	0.249**	1.000	−0.028	−0.073	0.136	0.204*
Pre CSC	0.097	−0.660***	0.217*	−0.171	−0.028	1.000	0.489***	−0.147	0.073
Post CSC	0.030	0.253**	0.059	−0.122	−0.073	0.489***	1.000	0.044	0.096
Pre IDH	−0.251**	0.213*	−0.637***	0.529***	0.136	−0.147	0.044	1.000	0.276**
Post IDH	0.089	0.043	0.511***	0.140	0.204*	0.073	0.096	0.276**	1.000
	**⊿SSC**	**XLIF levels**	**Cage Position**	**Cage Obliquity**					
⊿SSC	1.000	−0.129	−0.269**	0.037					
XLIF levels	−0.129	1.000	0.218*	0.126					
Cage Position	−0.269**	0.218*	1.000	0.037					
Cage Obliquity	0.037	0.126	0.037	1.000					

*p < 0.05, **< 0.01, ***< 0.001 indicates significant differences between groups.

## Discussion

Various formulas have been reported to plan the corrective surgery for ASD. The most respected formula is that proposed by Schwab, but his formula was based on analysis of cases 60 years old, and the applicability of his formula to Japanese patients is often questioned due to the differences in body type between Westerners and Japanese^18–21^.

The first report of the corrective properties that XLIF has on alignment was reported by Pimenta, who described the insertion of the cages into the relatively rigid vertebral lateral rim after anterior disc dissection^22^. Especially in cases of lumbar degenerative scoliosis accompanied by osteophytes, the narrowed disc space can be accessed after resection of the osteophytes with dedicated bone chisels. Insertion of the large XLIF cage into the intervertebral disc naturally corrects the segmental coronal alignment. Restoration of lumbar lordosis is also important in surgeries to correct ASD, and LLIF can correct sagittal deformities with larger lateral interbody cages. Regarding the installation position of cages, Kepler and colleagues reported that segmental lordosis was obtained by putting the cage in the anterior position. They reported significant lordosis correction with a mean segmental increase of 3.7° at levels in which XLIF cages were inserted, which is similar to the correction angle found in the current study^23^. Several studies have reported that the more the cage is positioned anteriorly, the greater the lordosis obtained^23,24^. From the correlation analysis, our current study also suggests that the more anterior the cage position, the greater the lordosis that is obtained. On the other hand, there is also study that reports cage position is not a factor^7^, and the effect of cage position is still under discussion. According to the systematic review by Phan *et al*. published in 2015^25^, only three studies examined changes in coronal segmental Cobb angles. They reported that the average coronal segmental Cobb angles changed from 3.6° preoperatively to 1.1 ° after surgery. The average preoperative and postoperative coronal regional Cobb angles were 19.1° and 10.0°, respectively. Sagittal segmental Cobb and regional lumbar lordosis also significantly improved after the XLIF surgery. Furthermore, previous reports demonstrated that although XLIF significantly improves lumbar scoliosis and segmental lordosis, there was no effect on global lordosis. These results were different from our study. We think that global lordosis is influenced by the number of levels at which XLIF was performed and by the angle of preoperative global lordosis^14,17^. Unlike previous reports, many patients in our series had 3-level fusions carried out and had relatively small preoperative lumbar lordosis. However, these reports did not evaluate spinal alignment immediately after XLIF surgery.

When the alignment after XLIF surgery was analyzed by CT-MPR, XLIF surgery improved segmental coronal, sagittal alignment, and VBR with an average correction angle of 2.0°, 4.0°, 1.4° respectively, at the instrumented segments where XLIF cages were inserted. There was no statistical difference in the sagittal segmental Cobb angle obtained for each XLIF level. Despite the increase in intervertebral disc height obtained by XLIF surgery, possible reasons for the low restoration of lordosis at the L4/5 level include arthropathic changes of the posterior components of L4/5 (the ankylosis of the facet joint), selection of the cage size, and position of the cage. Each of these points need to be studied in the future to address this issue.

For kyphotic cases, it is difficult to obtain the proposed 10° lordosis in the lower lumbar spine with XLIF alone. We propose that this is an important finding because XLIF alone cannot greatly change the lordosis at the L4/5 level. Therefore, additional correction procedures including osteotomies and compression techniques from the posterior are believed to be necessary.

There are several limitations in this study. First, this was a retrospective study, so a future prospective study with a more detailed evaluation should be considered to determine the best strategy for correction of spinal alignment. Second, there is a wide spectrum of ASD cases that may be difficult to analyze as one group. Mild ASD cases have deformities mainly limited to the lateral side of the intervertebral disc whereas cases with severe kyphoscoliosis not only have deformity in the posterior components but may also have contraction of the anterior longitudinal ligaments as well as osteophytes and ossifications of the intervertebral discs. Many of our patients were kyphotic with a mean SVA of 181 mm in which sagittal alignment is markedly shifted anteriorly, and the possibility that this affected our results cannot be denied. Third, CT can only be obtained in the supine position and cannot evaluate standing alignment. Low-dose EOS system^®^ (EOS Imaging, Paris, France) is considered an innovative slot-scanning radiograph system that provides valuable data for spinal surgery^26^. It can obtain radiographic images even when the patient is standing, sitting or squatting. We think that it is useful for evaluation in standing position if we use this. Furthermore, to properly evaluate the multiple factors that affect alignment, future studies will require more cases so that the relationship between cage position and the various Cobb angles as well as intervertebral disc heights can be examined for each level. Finally, most cages used in this study were 10° cages, but there were some 15° cages and the cage heights were also different. The selection of the cage angle as well as height may also be affecting the results.

## Conclusion

The XLIF technique does improve scoliosis and segmental kyphosis in patients with ASD. However, there is a limit to the sagittal and coronal correction that can be gained with XLIF alone. To further evaluate the effect of XLIF on sagittal balance, long-term follow-up with a larger cohort will be required.

## Patients and Methods

### Ethics

The study protocol was reviewed and approved by The Committee on Ethics and the Institutional Review Board of Tokai University School of Medicine (18R-253), the House Clinical Study Committee, and Profit reciprocity Committee. With this study being retrospective, the requirement for informed consent was not deemed necessary.

### Included patients

Patients were eligible for study enrollment if they were over 50 years old and were diagnosed with ASD that was unresponsive to conservative treatment of at least 6 months. Before surgery, all patients were radiologically evaluated with full length postero-anterior and lateral standing x-rays and CT-MPR. Generally, surgical treatment of thoracolumbar kyphoscoliosis is selected according to whether the deformity is mobile or fixed. Thus, we performed the supine bending such as lateral flexion, extension, and traction radiographs to examine the flexibility of the spinal deformity. CT can also be used to evaluate the status of the facet joints. Facet joint degeneration on the approach side was evaluated using the CT grading system^27^. In this series, patients with severe fixed anterior column that required three-column osteotomy were excluded.

Patient characteristics and operative details are given in Table 7. We conducted our retrospective review using a retrospective cohort. The study included ASD patients who underwent LLIF procedure using XLIF PEEK cages (NuVasive Inc., San Diego, CA, USA) at a single institute from January 2016 to December 2018. In total, 38 patients (aged 70.7 ± 7.8 years; 3 males and 35 females) who underwent XLIF with a transpsoas approach in a minimally invasive fashion, at one or more intervertebral levels, were enrolled in this study. Five of the 38 patients had degenerative kyphosis (DK), while the remaining 33 patients had degenerative kyphoscoliosis (DKS). The patients diagnosed with ASD were classified according to the SRS Schwab classification system^28^. In terms of the type of curve, 24 cases (63.2%) had a Lumbar curve (Type L), 1 case (2.6%) had a Double curve (Type D), and 13 cases (34.2%) did not have a curve in the coronal plane (Type N), but only a deformity in the sagittal plane. The average grade of facet joint degeneration on the approach side was 1.4; 17 (14.9%) levels were not affected by facet joint degeneration (grade 0), 55 (48.2%) levels presented with grade 1, 24 (21.1%) levels with grade 2, and 18 (15.8%) levels with grade 3. XLIF was performed across an average of 3.0 levels per patient on intervertebral levels L1–2 to L4–5.

**Table 7:** Demographic and clinical data.

Patients (*n*)	38
Age (*years*)	70.7 ± 7.8
Female	35 (92.1%)
**Diagnosis:**	
DK	5
DKS	33
**Type of curve: SRS-Schwab Classification**	
T	0 (0%)
L	24 (63.2%)
D	1 (2.6%)
N	13 (34.2%)
**Facet degeneration**	
Grade: 0/1/2/3	17/55/24/18
Approach side of XLIF (Left side)	25 (65.8%)
Spine levels	114
L1-2	15 (13.2%)
L2-3	34 (29.8%)
L3-4	35 (30.7%)
L4-5	30 (26.3%)
Blood loss (*cc*)	142.0 ± 80.5
Surgery duration (*min*)	147.3 ± 138.5

DK: Degenerative kyphosis.

DKS: Degenerative kyphoscoliosis.

### Operative data

The operations were performed by four spine surgeons. A two-stage surgery was adopted: the first stage was the minimally invasive multilevel XLIFs via transpsoas approach, using the 10 or 15-degree lordotic XLIF cages. The second stage was the posterior corrective fusion with TLIF performed at L5-S1 and/or L4-L5 and posterior instrumentation using pedicle screws.

XLIF was performed through a right-sided approach in 13 patients and through a left-sided approach in 25 patients. Basically, in cases without significant coronal deformity, a left-sided approach was performed. In many cases, we performed the XLIFs on the concave side of the scoliosis with the hinge in the radiolucent table flexed opposite the curve to help reduce the scoliosis. As a result, 19 (76%) out of 25 patients except type N group have approached from the concave side.

Interbody fusion was completed using the XLIF technique as described by Ozgur *et al*.^7^. Briefly, the patient is positioned in the lateral decubitus position with the concave side of the lumbar coronal curve facing up. This facilitates access to the largest number of disc spaces with a relatively small incision. Blunt dissection is then used to access the disc spaces under fluoroscopic guidance. After removal of the disc material with a rongeur, a Cobb elevator was advanced gently under fluoroscopy guidance along the endplates to release the contralateral annulus. Cage size trials were followed by additional disc curettage and rasping of the endplates. All cages were inserted using two containment sliders to protect the endplates and to keep graft material inside the cage. For all patients, side-to side cage size was decided by width of the endplates at that level based on intraoperative fluoroscopic guidance, and titanium cages of a standard 18-mm width were used. The maximum distraction achieved during discectomy using the trial inserts provided guidance as to the height of the cage. The choice of these XLIF cages (CoRoent XL; NuVasive Inc. San Diego, CA, USA) was decided by the surgeon. Cage lengths ranged from 40 to 55 mm and heights from 8 to 12 mm.

For graft material, artificial bone material comprising hydroxyapatite and collagen (Refit; HOYA Techno-surgical, Tokyo, Japan) soaked in autologous bone marrow aspirate.

### Radiological parameters

Radiographic assessment was performed preoperatively and postoperatively (within 7 days) using CT-MPR in contiguous 1-mm slices. CT-MPR was performed with the patients in spine position.

**Figure 2:**
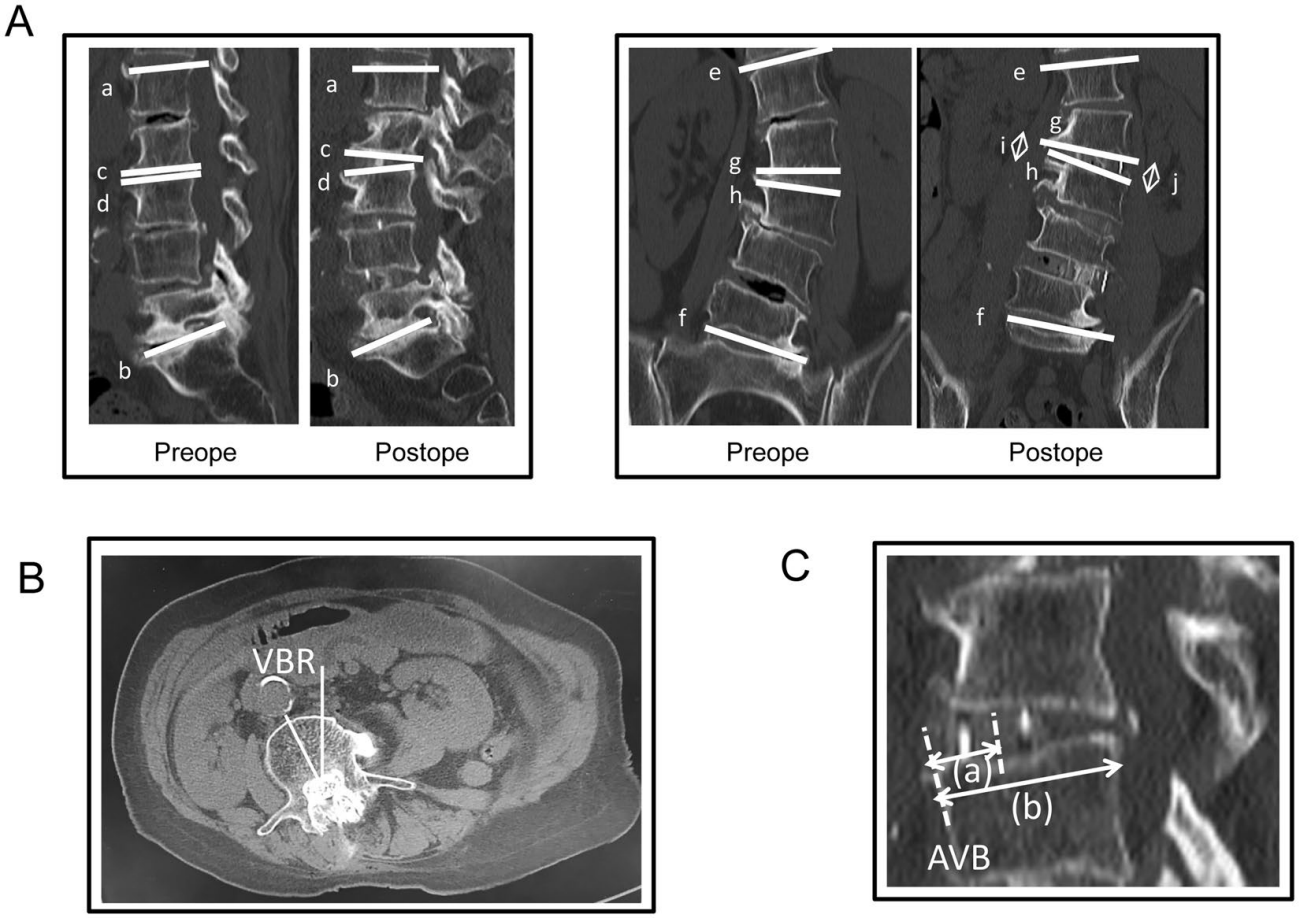
(**A**) Diagram showing the measurement of regional lumbar lordosis (L1-L5) (ab), sagittal segmental Cobb angle (cd), coronal regional Cobb angle (L1-L5) (ef), coronal segmental Cobb angle (gh), and disc height (arrow; i or j). (**B**) The rotation for each curve was measured from the pre- and postoperative axial CT. The measured vertebral body rotation (VBR) was expressed relative to a vertical line which corresponded to VBR of 0°. (**C**) A midsagittal CT scan showing an XLIF cage implanted into the disc space. The cage center is located by the midpoint between the anterior and posterior radiomarkers of the cage. The arrow (a) indicates the distance between the anterior vertebral border (AVB) of the inferior endplate and the center of the cage. The arrow (b) indicates the anteroposterior width of the inferior end plate. Cage position = 100 × a/b (%).

The radiographic parameter studied were coronal segmental Cobb angle, coronal regional Cobb angle (L1-L5), coronal disc height, sagittal segmental Cobb angle, and regional lumbar lordosis (L1-L5) (Fig. 2A). We also examined the pre and postoperative values for VBR (Fig. 2B). The rotation for each curve was measured from the pre- and postoperative axial CT-MPR using the methods described by Aaro and Dahlborn^29^.

The obtained sagittal segmental Cobb angle, coronal segmental Cobb angle, and disc height were expressed as ⊿ sagittal segmental Cobb angle (post-pre), ⊿ coronal segmental Cobb angle (post-pre), and ⊿ Intervertebral disc height (post-pre), respectively. Disc height was measured as an average of left and right disc heights.

We also used standard measurements reported elsewhere^30^ to assess coronal Cobb angle, sagittal vertical axis (SVA), lumbar lordosis (LL; T12–S1), thoracic kyphosis (TK; T5–12), pelvic incidence (PI), pelvic tilt (PT), and sacral slope (SS) by x-ray of the whole spine in standing position. The PI was measured as the angle between a line drawn perpendicular to the sacral end plate at its midpoint and a line drawn from the midpoint of the sacral end plate to the midpoint of the femoral head axis. The LL is the sagittal cobb angle measured between the superior end plate of T12 and the superior end plate of S1^31^.

For CT evaluation, cases in which angle measurements were not possible due to a high degree of kyphosis were excluded from this study.

We also examined whether the position of the cage correlates with each evaluation angle. Cage positioning was evaluated using pre and postoperative CT scans in the midsagittal plane. The distance between the anterior vertebral border (AVB) of the inferior end plate and the center of the cage was measured and normalised to the AP width of the inferior end plate. The center of the cage was defined as the midpoint between the anterior and posterior radio-markers of the cage (Fig. 2C).

### Statistical analysis

Statistical analyses were performed using IBM SPSS Statistics version 20.0 (IBM Corp., Armonk, NY). All values are expressed as mean ± standard deviation. An analysis of variance with a posthoc test (Mann–Whitney *U* test) was used for comparisons. The correlations between cage position and radiological parameters were analyzed using Spearman’s product-moment correlation coefficient.

To identify the minimum number of participants required for adequate statistical power, we used the G-Power Analysis software program (G Power 3.1.9, University of Düsseldorf, Germany, http://www.gpower.hhu.de/)^32^.

A power analysis performed to calculate the minimum sample size necessary to detect a difference between two dependent groups (calculated with Cohen’s d = 0.5, alpha = 0.05, power = 0.8) indicated a required sample size of 34 participants. A power analysis performed to calculate the minimum sample size necessary to detect a correlation (calculated with Correlation *ρ* H1 = 0.3, alpha = 0.05, power = 0.8) indicated a required total sample size of 84 participants. For all statistical analyses, the type 1 error was set at 5% and *p* < 0.05 was considered significant.
